# Remnant cholesterol shows inverse and nonlinear associations with leukocyte telomere length and serum α-Klotho, mediated by inflammation and oxidative stress

**DOI:** 10.3389/fendo.2025.1700349

**Published:** 2025-11-17

**Authors:** Baodi Xing, Jie Yu, Yiwen Liu, Qi Gao, Xinyue Chen, Shuli He, Fan Ping, Lingling Xu, Wei Li, Huabing Zhang, Yuxiu Li

**Affiliations:** 1 Department of Endocrinology, Key Laboratory of Endocrinology of National Health Commission, Translation Medicine Center, Peking Union Medical College Hospital, Chinese Academy of Medical Sciences and Peking Union Medical College, Beijing, China; 2 Department of Nutrition, Peking Union Medical College Hospital, Chinese Academy of Medical Sciences and Peking Union Medical College, Beijing, China

**Keywords:** remnant cholesterol, biological aging, leukocyte telomere length, serum α-Klotho, inflammation, oxidative stress

## Abstract

**Background:**

Remnant cholesterol (RC) has been implicated in cardiovascular and metabolic disorders, but its direct associations with biological aging remain unclear. We aimed to examine the relationship between RC and two established aging biomarkers, leukocyte telomere length (LTL) and serum α−Klotho, and to evaluate the potential mediating roles of inflammation and oxidative stress.

**Methods:**

This cross-sectional analysis included 1052 adults from a rural cohort in northern China. Linear regressions and restricted cubic splines (RCS) assessed linear and nonlinear relationships. Mediation models explored the mediating effect of inflammation (TNFα, IL-6, IL-1β) and oxidative stress markers (SOD, 8-OHdG).

**Results:**

Participants with higher RC levels had lower LTL and α-Klotho levels, along with higher levels of TNFα, IL-6, IL-1β, and SOD. In adjusted linear regression, RC showed negative associations with both LTL and α-Klotho (β[95%CI]: -0.177[-0.262, -0.091] and -0.045[-0.066, -0.024]), independent of conventional lipid profiles. Both relationships were nonlinear (*P* for nonlinear = 0.001 for LTL; 0.019 for α-Klotho). For LTL, the inverse association was confined to 0.65-1.42 mmol/L (no significant associations outside this range), while for α-Klotho it was observed only below 1.37 mmol/L (no associations above it). TNFα and IL-6 partially mediated the relationship between RC and LTL (17.78% and 14.12%, respectively); while SOD partially mediated the association between RC and α-Klotho (58.18%).

**Conclusions:**

RC is inversely and nonlinearly associated with both LTL and α-Klotho, independent of traditional lipid profiles, with inflammation and oxidative stress as partial mediators. From an aging perspective, these cross-sectional findings support increased attention to early RC management alongside traditional lipids.

## 1 Introduction

As the global population continues to age, age-related health issues have emerged as major public health challenges worldwide (1). Among the contributing factors, abnormal lipid metabolism has been recognized as a key risk factor for numerous aging-associated diseases, including type 2 diabetes mellitus (T2DM), atherosclerosis, and cancers (2). Targeting lipid metabolism is thus considered a promising strategy for clinical interventions to delay aging.

Remnant cholesterol (RC), also known as triglyceride-rich lipoprotein cholesterol, refers to the cholesterol content within very low-density lipoproteins (VLDL) and chylomicron remnants, excluding both high-density lipoproteins (HDL-C) and low-density lipoproteins (LDL-C). Substantive evidence indicates that RC is more strongly associated with cardiovascular disease (CVD) and mortality than traditional lipid markers, such as LDL-C (3, 4). Moreover, several large-scale studies have shown that elevated RC levels predict an increased risk of T2DM and premature mortality (5, 6). Despite its emerging importance in chronic diseases, the role of RC in the biological aging process remains largely unexplored.

Telomeres are specialized DNA-protein structures at the ends of eukaryotic chromosomes that preserve chromosomal stability and genomic integrity. Telomere length progressively shortens with each cell division and during the aging process, and telomere attrition has been linked to increased risk of various metabolic and age-related disorders (7, 8). α−Klotho is a single-pass transmembrane protein encoded by the Klotho gene. Its extracellular domain can be shed to generate a soluble form that circulates in serum (serum α−Klotho), which is widely recognized for its anti−aging functions (9). Mice with α-Klotho deficiency exhibit a markedly shortened lifespan and display premature aging phenotypes, such as atherosclerosis, muscle wasting, and osteoporosis. Conversely, Klotho overexpression extends lifespan (10). In humans, serum α-Klotho declines with age and relates to adverse outcomes (11–13); moreover, better cardiovascular health (Life’s Essential 8) has been linked to higher circulating α-Klotho, highlighting its cardiometabolic relevance (14). As such, leukocyte telomere length (LTL) and serum α-Klotho levels are commonly used biomarkers of biological aging and to evaluate anti-aging interventions (7, 9). However, the association between RC and these aging markers remains poorly defined.

It is well established that chronic low-grade inflammation and oxidative stress are key mechanisms by which RC promotes the development of CVD and T2DM (15–17). These processes also contribute significantly to the biological aging process and have been implicated in both telomere shortening and α-Klotho depletion (18–20). Accordingly, we hypothesize that elevated RC may be associated with decreased LTL and α-Klotho levels through inflammatory and oxidative pathways. Nonetheless, current evidence supporting this hypothesis is limited. To date, only one study in U.S. adults has reported a potential negative association between RC and serum α-Klotho (21); however, it lacked a mechanistic evaluation and has not been replicated in other ethnic populations. Furthermore, research investigating the link between RC and LTL is scarce.

Therefore, this study aims to investigate the relationship between RC and two established aging biomarkers, LTL and serum α-Klotho, in a Chinese cohort. Additionally, we seek to elucidate the potential mediating roles of inflammation and oxidative stress in these associations, thereby contributing to a better understanding of RC’s role in aging and its potential as a clinical target for aging-related interventions.

## 2 Materials and methods

### 2.1 Study population

This cross-sectional study was conducted using data from a long-term, ongoing natural population cohort in the rural districts of Changping, Beijing, China. The cohort, initiated in March 2014 (22), included adults aged 18–84 years and aimed to investigate the relationships among nutrition, metabolism, and aging. Participants were enrolled on a rolling basis, with follow-up assessments conducted every 1 to 3 years for both initial and newly recruited subjects.

A total of 1275 participants were initially enrolled, all of whom voluntarily provided written informed consent. Individuals were excluded based on the following criteria: (i) presence of severe cardiovascular diseases, liver or renal insufficiency (n=77); (ii) use of dietary supplements or drugs that may influence serum lipids (e.g., lipid-lowering medications) (n=46); and (iii) missing measurements of LTL, serum α-Klotho, and serum lipids (n=100). Ultimately, 1052 eligible participants were included in the final analysis.

### 2.2 General characteristics and dietary information collection

Face-to-face interviews were conducted by uniformly trained physicians using a standardized questionnaire to collect baseline information from study participants, including age, sex, dietary information, medical history, and medication history. Dietary information was collected using a 24-hour food recall, as previously documented (22).

### 2.3 Anthropometric assessment

All participants were measured for height, weight, waist circumference (WC), hip circumference (HC), systolic blood pressure (SBP), and diastolic blood pressure (DBP), as detailed in previous studies (23). Body mass index (BMI) was calculated as body weight divided by height squared (kg/m^2^). The waist-to-hip ratio (WHR) was the ratio of WC to HC.

### 2.4 Biochemical analysis

Venous blood samples were collected after an overnight fast of more than 10 hours, to measure indicators correlated to serum glucose and lipids. Fasting plasma glucose (FPG) was measured using an oxidase method, while fasting insulin (F-INS) levels were determined by chemiluminescence assays. Glycosylated hemoglobin (HbA1c) was analyzed using high-performance liquid chromatography, with intra-assay and inter-assay coefficients of variation below 3% and 10%, respectively. Lipid profiles, involving TG, TC, LDL-C, and HDL-C, were evaluated by an automated analyzer. The LDL-C levels were calculated based on TC, HDL-C, and TG levels according to the Friedewald formula unless TG was significantly elevated (>4 mmol/L). RC was estimated as TC minus HDL-C minus LDL-C (4, 24). Liver and Renal function indicators, including alanine aminotransferase (ALT), aspartate aminotransferase (AST), serum creatinine (sCr), and serum uric acid (sUA), were also measured using the above method. The estimated glomerular filtration rate (eGFR) was assessed using the Chronic Kidney Disease Epidemiology Collaboration equation (25). Additionally, insulin sensitivity and islet β-cell function were evaluated using two fasting indices–homeostatic model assessment of insulin resistance (HOMA-IR) and β-cell function (HOMA-β), as previously described (26).

### 2.5 LTL and α-Klotho measurements

Peripheral blood LTL analysis has been described in detail previously (20). Briefly, the QIAamp DNA blood mid kit (Qiagen, Hilden, Germany) was applied to extract genomic DNA in leukocytes. Purified DNA samples were diluted and quantified using a NanoDrop 1000 spectrophotometer (Thermo Fisher Scientific, Wilmington, DE, USA). LTL was represented as the relative ratio of telomere repeat copy number to the single copy number (T/S) using novel monochrome multiplex quantitative PCR. The within-plate and between-plate CVs were 18% and 7%, respectively. Due to non-batch measurement, the z scores standardized LTL (z-LTL) was computed and applied for all analyses to minimize the impact of potential batch shift (27).

Serum α-Klotho was measured by a commercially available ELISA kit produced by IBL International, Japan. The assay had a sensitivity of 6.15 pg/mL. The measurement range for serum α-Klotho was 93.75 to 6000 pg/mL, with an intra-assay coefficient of variation (CV) of 3.1% and an inter-assay CV of 6.9% (28).

### 2.6 Measurements of inflammation and oxidative stress indicators

Serum concentrations of inflammatory cytokines, including tumor necrosis factor α (TNFα), interleukin-6 (IL-6), and interleukin-1β (IL-1β), were measured using Luminex^®^ X-MAP technology (Luminex Corp., Austin, TX, USA). Oxidative stress markers, including superoxide dismutase (SOD) activity and 8-hydroxy-2-deoxyguanosine (8-OHdG), were assessed via enzyme-linked immunosorbent assay (ELISA, Cloud-Clone Corp., Houston, TX, USA). Sample processing and data analysis were conducted in strict accordance with the manufacturer’s protocols.

### 2.7 Statistical analysis

Continuous variables were presented as means with standard deviations (SD) for normally distributed data or as medians with interquartile ranges (IQR) for skewed data. Categorical variables were presented as frequencies with percentages. Baseline characteristics across RC quartiles were compared using one-way ANOVA for normally distributed continuous variables (Welch’s ANOVA when variances were unequal), the Kruskal-Wallis test for skewed continuous variables, and the chi-square test for categorical variables. Normality was assessed using the Shapiro-Wilk test and inspection of histograms and Q-Q plots; homogeneity of variances was evaluated using Levene’s test. For variables with overall differences (*P*<0.05), pairwise comparisons used Tukey’s HSD after ANOVA (Games-Howell after Welch’s ANOVA) or Dunn’s test with Bonferroni correction after the Kruskal-Wallis test.

Due to the non-normal distribution, α-Klotho levels were log-transformed to achieve normality before analysis. Univariate linear regression was employed to assess the relationship between traditional lipid parameters, RC, and aging markers. Multivariate linear regression analyses were then conducted with RC treated as both a continuous and a categorical variable (quartiles, using Q1 as the reference), to assess its relationship with LTL and α-Klotho. Four main models were constructed for potential covariate adjustments: Model 1 was adjusted for age and sex; Model 2 further incorporated BMI, WHR, ALT, AST, eGFR, SBP, DBP, HbA1c, FPG, TG, HDL-C, LDL-C, and UA; Model 3 additionally accounted for total energy intake based on Model 2; and Model 4 expanded on Model 3 by including TNF-α, IL-6, IL-1β, SOD, and 8-OHdG. Multicollinearity among covariates was evaluated using variance inflation factors (VIFs), with values >10 indicating pronounced multicollinearity. And all variables in our models had VIFs <5. Nonlinear relationships between RC and both aging biomarkers were explored with restricted cubic splines (RCS) using knots at the 5th, 35th, 65th, and 95th percentiles, with analysis and visualization via the plotRCS package. When a nonlinear pattern was detected, threshold analysis with the segmented package was used to identify inflection points, and piecewise linear regression was subsequently fitted based on those cut points.

Missing values of covariates in the model were imputed with the missForest package in R (a random forest–based method) (8). The algorithm accommodates both continuous and categorical data and is robust to nonlinearity and outliers, with strong accuracy and reliability. The number and percentage of missing values for each covariate are provided in **Supplementary Table S1**.

For the mechanistic analysis, PROCESS macro Version 3.4 (29) was performed to investigate whether inflammation and oxidation markers play mediation roles among these relationships. Statistical significance of mediating effects was admitted if the 95% confidence interval did not include zero, as previously described (23).

Statistical analyses were performed by SPSS Windows, version 26.0 (IBM Corp., Chicago, IL, USA) and R software (version 4.2). A two-sided *P*-value<0.05 was considered statistically significant.

## 3 Results

### 3.1 Baseline characteristics of the study population between different groups

The average age of the overall study population was 55.68 ± 10.85 years, and 63.5% were female. Compared to participants in the lower RC level groups, those in the higher RC level groups exhibited poorer metabolic profiles, characterized by elevated WC, WHR, ALT, AST, TC, TG, LDL-C, FPG, HOMA-IR, HOMA-β, as well as decreased eGFR and HDL-C (all *P <*0.05). Regarding aging biomarkers and inflammatory/oxidative stress indicators, individuals in the higher RC quartiles had significantly lower levels of z-LTL and serum α-Klotho, whereas TNF-α, IL-6, IL-1β, and SOD levels were significantly higher. Additionally, significant differences were also observed across RC groups in terms of age, SBP, DBP, HbA1c, and total energy intake (all *P <*0.05). There were no significant differences between groups for sex, BMI, and 8-OHdG (all *P* > 0.05) (**Table 1**).

**Table 1:** Baseline characteristics of participants across RC quartiles.

Indicators	Overall (n=1052)	Q1 (n=270) (≤0.530)	Q2 (n=259) (0.530~1.020)	Q3 (n=262) (1.020~1.380)	Q4 (n=261) (>1.380)	*P*
Age (y)	55.68 ± 10.85	56.34 ± 11.50	57.54 ± 10.73	53.02 ± 10.69^ab^	55.79 ± 9.97^bc^	<0.001*
Female	638 (63.5)	148 (58.3)	158 (62.7)	163 (65.7)	169 (67.6)	0.144
Male	366 (36.5)	106 (41.7)	94 (37.3)	85 (34.3)	81 (32.4)	
BMI (kg/m^2^)	26.41 ± 7.83	26.51 ± 14.20	26.36 ± 3.57	26.25 ± 3.91	26.52 ± 3.78	0.977
WC (cm)	89.00 ± 10.60	88.49 ± 11.27	88.77 ± 9.97	88.45 ± 11.53	90.29 ± 9.44^abc^	0.028*
WHR	0.93 (0.89, 0.95)	0.90 (0.84, 0.93)	0.91 (0.88, 0.95)	0.95 (0.93, 0.96)	0.95 (0.93, 0.96)	<0.001*
SBP (mmHg)	129.78 ± 17.27	131.07 ± 16.96	131.80 ± 17.25	127.55 ± 16.80^ab^	128.66 ± 17.80	0.018*
DBP (mmHg)	78.20 ± 10.82	78.70 ± 10.36	80.11 ± 11.11	76.77 ± 10.58^ab^	77.19 ± 10.97^b^	0.002*
ALT (U/L)	20.80 (15.45, 28.02)	18.00 (14.00, 23.00)	19.00 (15.00, 25.25)	22.85 (17.08, 31.45)	24.00 (17.85, 34.98)^ab^	<0.001*
AST (U/L)	21.95 (18.00, 26.00)	21.00 (18.00, 24.00)	21.00 (18.00, 24.65)	22.45 (19.10, 26.42)	23.00 (19.20, 27.82)^ab^	<0.001*
eGFR (mL/min/1.73m^2^)	94.21 ± 19.27	99.08 ± 20.11	93.70 ± 18.59^a^	93.65 ± 18.12^a^	90.35 ± 19.28^a^	<0.001*
sUA (umol/L)	297.23 ± 79.19	290.18 ± 71.87	305.15 ± 78.58^a^	288.12 ± 79.40^b^	305.45 ± 85.26^c^	0.014*
TC (mmol/L)	5.13 ± 1.09	4.53 ± 0.90	4.70 ± 0.96	5.13 ± 0.68^ab^	6.16 ± 0.97^abc^	<0.001*
TG (mmol/L)	1.39 (0.97, 2.06)	0.97 (0.71, 1.22)	1.56 (1.10, 2.00)	1.35 (0.99, 1.98)^a^	2.24 (1.55, 3.71)^abc^	<0.001*
HDL-C (mmol/L)	1.27 ± 0.35	1.34 ± 0.28	1.26 ± 0.50	1.24 ± 0.27^a^	1.26 ± 0.29^a^	0.007*
LDL-C (mmol/L)	2.85 ± 0.76	2.81 ± 0.76	2.77 ± 0.79	2.65 ± 0.57	3.18 ± 0.80^abc^	<0.001*
FPG (mmol/L)	6.05 (5.48, 7.30)	6.00 (5.30, 7.50)	6.10 (5.40, 8.00)^a^	5.92 (5.51, 6.60)^b^	6.22 (5.63, 7.17)^ac^	0.025*
HbA1c (%)	5.70 (5.40, 6.53)	5.80 (5.40, 6.70)	5.80 (5.40, 7.00)	5.60 (5.30, 6.10)^ab^	5.80 (5.43, 6.40)^c^	<0.001*
HOMA-IR	2.56 (1.67, 4.02)	1.92 (1.34, 3.17)	2.75 (1.71, 4.03)^a^	2.69 (1.78, 4.01)^a^	2.97 (2.07, 4.97)^a^	<0.001*
HOMA-β	68.63 (41.85, 100.10)	53.18 (33.81, 81.21)	67.87 (38.85, 101.78)	75.36 (49.64, 105.90)^ab^	71.58 (50.04, 109.79)^a^	<0.001*
Energy (kcal/d)	1379.71 (1037.81, 1777.43)	1271.09 (963.95, 1698.50)	1325.15 (986.53, 1674.15)	1452.36 (1126.87, 1809.49)^a^	1440.59 (1094.07, 1811.41)	0.002*
z-LTL	0.02 ± 0.58	0.15 ± 0.54	0.08 ± 0.59	-0.06 ± 0.61^ab^	-0.10 ± 0.53^ab^	<0.001*
α-Klotho (pg/mL)	809.24 ± 264.64	884.81 ± 294.93	816.11 ± 271.04^a^	759.34 ± 228.84^a^	775.02 ± 241.26^a^	<0.001*
TNFa (pg/mL)	5.57 (3.64, 8.18)	4.95 (3.28, 6.71)	5.31 (3.49, 7.30)	6.13 (4.08, 10.76)^ab^	6.31 (3.75, 10.04)^ab^	<0.001*
IL-1β (pg/mL)	0.80 (0.27, 4.02)	0.33 (0.22, 0.85)	0.44 (0.23, 1.42)	2.79 (0.73, 20.91)^ab^	1.80 (0.57, 10.03)^ab^	<0.001*
IL-6 (pg/mL)	0.67 (0.31, 1.81)	0.50 (0.28, 0.95)	0.64 (0.36, 1.29)	0.98 (0.35, 9.05)^ab^	0.76 (0.30, 2.69)^a^	<0.001*
SOD (U/mL)	18.21 (14.57, 59.03)	14.84 (14.21, 15.67)	15.07 (14.17, 24.57)	56.45 (42.59, 66.61)^ab^	54.71 (34.21, 63.68)^ab^	<0.001*
8-oHdG (ng/mL)	38.00 (26.87, 50.57)	38.87 (30.63, 47.16)	37.29 (27.75, 47.44)	40.74 (24.90, 55.06)	35.88 (21.71, 57.97)	0.483

BMI, body mass index; WC, waist circumference; WHR, waist-hip ratio; SBP, systolic blood pressure; DBP, diastolic blood pressure; ALT, alanine aminotransferase; AST, aspartate aminotransferase; sUA, serum uric acid; TC, total cholesterol; TG, triglyceride; HDL-C, high-density lipoprotein cholesterol; LDL-C, low-density lipoprotein cholesterol; FPG: fasting plasma glucose; HbA1c, glycated hemoglobin; HOMA-IR, homeostatic model assessment of insulin resistance; HOMA-β, homeostatic model assessment of β-cell function; z-LTL, z scores standardized LTL; TNFα, tumor necrosis factor α; IL-6, interleukin-6; IL-β, interleukin-1β; SOD, superoxide dismutase; 8-oHdG,8-hydroxy-2-deoxyguanosine.

*P<0.05 means statistical difference. ^a^represents a significant difference compared with the Q1 group; ^b^represents a significant difference compared with the Q2 group; ^c^represents a significant difference compared with the Q3 group.

### 3.2 The general linear regression of different lipid parameters and aging biomarkers

We first conducted univariate linear regression analyses to assess the associations between various lipid markers and two aging biomarkers. RC showed significant negative associations with both LTL and α-Klotho (β [95%CI]: -0.161 [-0.226, -0.095] and -0.029 [-0.045, -0.012], respectively; both *P <*0.001). For LTL, we also observed the reverse associations of TC, LDL-C, and TG, but all were weaker than that of RC. No traditional lipid marker was significantly associated with α-Klotho. (**Supplementary Table S2**).

### 3.3 The linear regression of RC and aging biomarkers in multivariate corrected models

Subsequently, RC was entered as a continuous variable into multiple linear regression models. In model 3, RC remained significantly and negatively associated with both LTL and α-Klotho, independent of age, sex, total energy intake, and various metabolic parameters, including traditional lipid markers (β [95%CI]: -0.177 [-0.262, -0.091] and -0.045[-0.066, -0.024], respectively; both *P <*0.05). When RC was categorized into quartiles, the negative associations with both aging markers became more pronounced across increasing RC quartiles, with the strongest inverse associations observed in the third quartile (Q3: β = -0.255 for LTL; β = -0.062 for α-Klotho; both *P*<0.05). Although the associations in the fourth quartile (Q4) were slightly weaker than those in Q3, they remained stronger than in the second quartile (Q2) (**Table 2**).

**Table 2:** The multiple linear regression between RC and LTL and serum α-klotho.

LTL	1mmol/L increment	Q1	Q2	Q3	Q4
model 1	-0.171(-0.236,-0.105)*	1(ref)	-0.061(-0.159,0.037)	-0.243(-0.342,-0.144)*	-0.263(-0.361,-0.164)*
model 2	-0.184(-0.269,-0.098)*	1(ref)	-0.038(-0.138,0.061)	-0.260(-0.362,-0.158)*	-0.235(-0.350,-0.120)*
model 3	-0.177(-0.262,-0.091)*	1(ref)	-0.038(-0.137,0.062)	-0.255(-0.358,-0.153)*	-0.230(-0.345,-0.114)*
model 4	-0.162(-0.250,-0.075)*	1(ref)	-0.033(-0.133,0.066)	-0.241(-0.345,-0.137)*	-0.217(-0.334,-0.010)*
α-Klotho	1mmol/L increment	Q1	Q2	Q3	Q4
model 1	-0.030(-0.046,-0.014)*	1(ref)	-0.032(-0.056,-0.007)*	-0.065(-0.090,-0.041)*	-0.054(-0.078,-0.029)*
model 2	-0.045(-0.066,-0.024)*	1(ref)	-0.030(-0.054,-0.005)*	-0.062(-0.087,-0.037)*	-0.058(-0.086,-0.030)*
model 3	-0.017 (-0.045,0.011)	1(ref)	-0.023(-0.048,0.002)	-0.043(-0.073,-0.013)*	-0.034(-0.069,-0.001)*
model 4	-0.017(-0.045,-0.011)	1(ref)	-0.030(-0.054,-0.005)*	-0.062(-0.087,-0.037)*	-0.058(-0.086,-0.030)*

Model 1 was adjusted for age and sex; model 2 was adjusted for BMI, WHR, ALT, AST, eGFR, SBP, DBP, HbA1c, FBG, TG, HDL-C, LDL-C, and sUA; model 3 was further adjusted for total energy intake based on model 2; and model 4 was further adjusted for TNF-α, IL-6, IL-1β, SOD, and 8-OHdG. The z-LTL and log-transformed α-klotho were analyzed in all models. *P<0.05 means statistical difference.

After further adjustment for inflammatory and oxidative stress markers in model 4, the association between RC (as a continuous variable) and LTL was attenuated but remained statistically significant (β =-0.162, *P*<0.05), while the association with α-Klotho was no longer significant (β =-0.017, *P* > 0.05). Similarly, the quartile-based inverse trends between RC and the two aging markers were also markedly attenuated (**Table 2**).

### 3.4 Non-linear relationship exploration between RC and two aging biomarkers through restricted cubic splines

**Figure 1:**
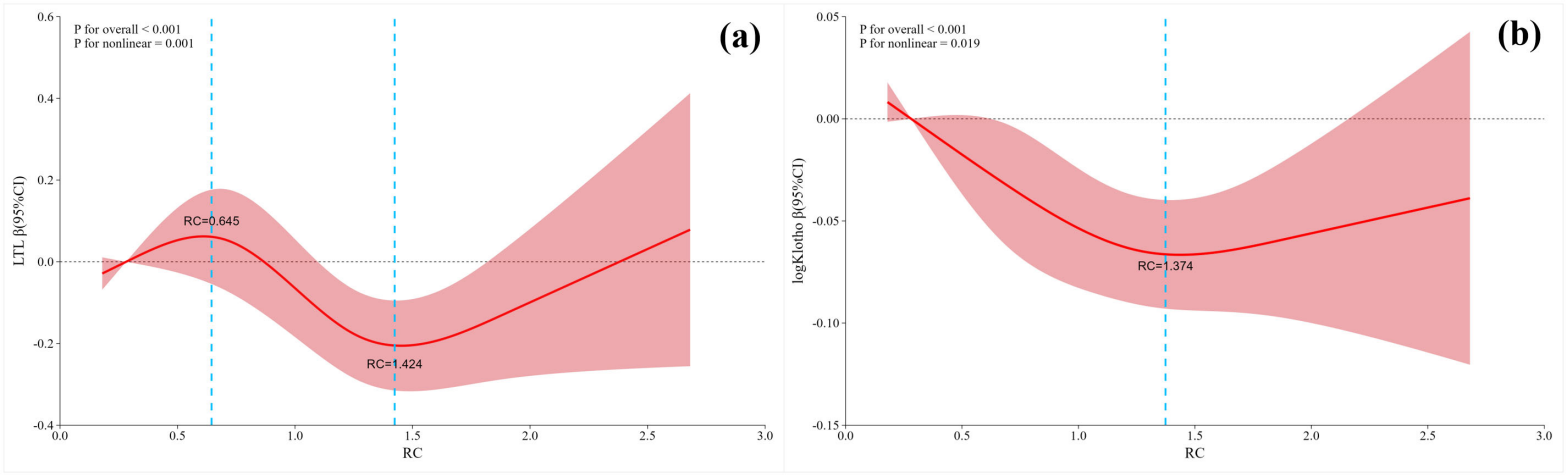
The nonlinear relationship between RC and two aging biomarkers. **(a)** RC and LTL; **(b)** RC and α-Klotho. The model was adjusted for age, sex, BMI, WHR, ALT, AST, eGFR, SBP, DBP, HbA1c, FPG, LDL-C, HDL-C, TG, sUA, and total energy intake.

Based on the results of linear regression using RC as a categorical variable, the associations between RC and both aging biomarkers appeared to be nonlinear. Therefore, RCS analyses were performed to further investigate the dose-response relationships. As shown in **Figure 1**, significant nonlinear associations were observed for both LTL and α-Klotho (*P* for nonlinear = 0.001 and 0.019, respectively). For LTL, two inflection points were identified at 0.645 and 1.424 mmol/L, whereas for α-Klotho a single inflection point was observed at 1.374 mmol/L.

Subsequent piecewise linear regression analyses revealed that RC was significantly negatively associated with LTL only within 0.645-1.424mmol/L and with α-Klotho only below 1.374mmol/L (β = -0.516 and -0.062, respectively; both *P*<0.05). No significant associations were observed when RC exceeded the respective cut points (all *P* > 0.05) (**Supplementary Table S3**).

### 3.5 Potential mechanism exploration of inflammation and oxidative stress

**Figure 2:**
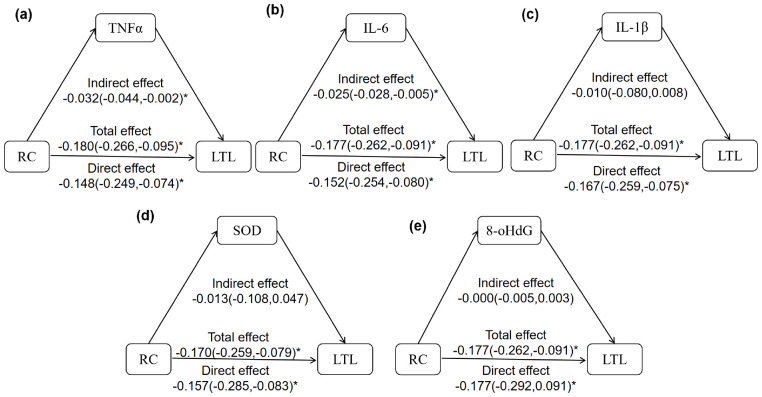
The mediation effect of inflammation and oxidative stress in the relationship between RC and LTL. **(a-e)** the mediation model of TNFα, IL-6, IL-1β, SOD, 8-OHdG on the correlation between RC and LTL. The model was adjusted for age, sex, BMI, WHR, ALT, AST, eGFR, SBP, DBP, HbA1c, FPG, LDL-C, HDL-C, TG, sUA, and total energy intake. The z-LTL and log-transformed TNFα, IL-6, IL-1β, SOD, and 8-OHdG were analyzed in the model. *P<0.05 means statistical difference.

**Figure 3:**
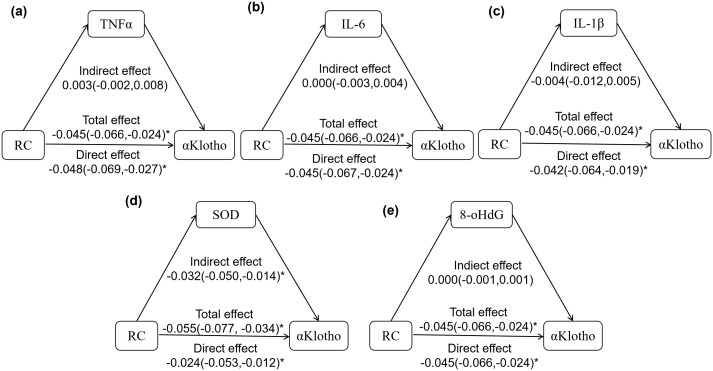
The mediation effect of inflammation and oxidative stress in the relationship between RC and α-Klotho protein. **(a–e)**: the mediation model of TNFα, IL-6, IL-1β, SOD, 8-OHdG on the correlation between RC and α-Klotho. The model was adjusted for age, sex, BMI, WHR, ALT, AST, eGFR, SBP, DBP, HbA1c, FPG, LDL-C, HDL-C, TG, sUA, and total energy intake. The log-transformed α-Klotho, TNFα, IL-6, IL-1β, SOD, and 8-OHdG were analyzed in the model. *P<0.05 means statistical difference.

Mediation analysis was conducted to assess the roles of five inflammatory and oxidative stress markers in the associations between RC and both aging biomarkers. As shown in **Figures 2a,b**, both TNF-α and IL-6 significantly mediated the inverse association between RC and LTL, with mediation proportions of 17.78% and 14.12%, respectively. In contrast, IL-1β, SOD, and 8-OHdG did not exhibit significant mediation effects in this pathway (**Figures 2c–e**). Regarding the association between RC and α-Klotho, only SOD showed a significant mediating effect, accounting for 58.18% of the total effect (**Figure 3d**). No significant mediation was observed for TNF-α, IL-1β, IL-6, or 8-OHdG in this relationship (**Figures 3a-c, e**).

## 4 Discussion

This cross-sectional study in a Chinese population identified inverse and nonlinear relationships between RC and two aging biomarkers, LTL and serum α-Klotho, independent of age, sex, and traditional metabolic risk factors, including triglycerides and LDL-C. Inverse associations were confined to specific RC ranges (LTL: 0.65-1.42 mmol/L; α-Klotho: below 1.37 mmol/L) and were not observed outside these ranges. Mediation analysis further revealed that inflammation, reflected by elevated TNF-α and IL-6, partially mediated the link between RC and LTL, while oxidative stress, indicated by elevated SOD, was involved in the association between RC and serum α-Klotho.

While traditional lipid parameters like LDL-C and TC have long been used to evaluate cardiovascular and metabolic risk, their relationship with aging is paradoxical. Emerging evidence has shown that higher LDL-C and TC levels are sometimes associated with longer telomere length and lower mortality risk in older adults—a phenomenon described as the “cholesterol paradox” (30–33). These findings highlight the complexity of lipid biology in aging and underscore the limitations of traditional lipids in capturing residual risk. RC, a cholesterol component of triglyceride-rich lipoprotein remnants, may better reflect underlying dyslipidemia and atherogenicity due to its higher cholesterol content per particle and greater propensity for arterial retention and inflammation (34). Numerous studies have confirmed the superior predictive value of RC in chronic conditions such as CVD, T2DM, and all-cause mortality (3–5). However, its relevance to biological aging has received limited attention.

In this study, individuals with elevated RC levels exhibited shorter LTL and lower α-Klotho protein levels, suggesting a potential link between higher RC and accelerated biological aging. Linear regression analysis confirmed negative associations between RC and both aging biomarkers, independent of age, sex, and multiple metabolic confounders, including traditional lipid markers (TG, LDL-C, HDL-C). Unlike prior studies that focused on aging-related diseases (5, 6), our study is the first to shift the focus directly to biological aging itself, using validated aging biomarkers as outcomes. We newly identified a negative association between RC and LTL, providing preliminary evidence that elevated RC may contribute to telomere shortening. Additionally, we confirmed and extended prior findings from a U.S. population by demonstrating a similar inverse relationship between RC and serum α-Klotho in a Chinese cohort (21). These consistent results across diverse populations suggest that RC may serve as a more sensitive lipid-related biomarker of aging than conventional lipids.

Importantly, our findings also demonstrated nonlinear associations between RC and aging biomarkers. For α-Klotho, the inverse association was evident only below 1.37 mmol/L. For LTL, a significant inverse association was observed within 0.65-1.42 mmol/L; however, in clinical lipid management, greater attention should be paid to elevated RC levels. Overall, these patterns mirror prior threshold-based relationships reported between RC and aging-related diseases (35–37). For example, RC showed an inverse L-shaped relationship with nonalcoholic fatty liver disease, with a turning point around 0.96 mmol/L (35). A similar nonlinear pattern was reported for stroke risk in a Chinese cohort, positive below 1.78 mmol/L and nonsignificant above (36). Another research on sarcopenia also supports the finding, with a turning point near 1.33 mmol/L (37). These patterns suggest a critical range below which RC exerts greater biological impact, while levels above the threshold may represent a saturation effect. In other words, beyond this “saturation point”, compensatory mechanisms may partially attenuate the detrimental effects of RC, implying a potential window for earlier RC management. However, reported RC thresholds vary across studies, likely due to the differences in populations, sample size, and modeling choices. Future research in large and diverse cohorts is needed to validate these thresholds and clarify their clinical relevance.

Mechanistically, mediation analyses confirmed that inflammation and oxidative stress, two well-established contributors to aging, partially explained the associations between RC and aging biomarkers. Specifically, TNF-α and IL-6 significantly mediated the relationship between RC and LTL. Chronic low-grade inflammation is known to suppress telomerase activity, impair telomere maintenance, and accelerate cellular senescence, with cumulative inflammatory burden, particularly involving TNF-α and IL-6, being inversely associated with LTL (18, 38). RC has also been shown to promote immune activation and vascular dysfunction, leading to sustained inflammatory responses (39). Our findings align with these mechanisms and provide further evidence that RC may be correlated with telomere shortening via inflammatory pathways.

In addition, SOD was identified as a significant mediator of the inverse association between RC and α-Klotho. As a key antioxidant, SOD can mitigate reactive oxygen (ROS) production by converting superoxide anions into hydrogen peroxide. Elevated SOD levels are generally considered an adaptive protective response (40). We observed that individuals with higher α-Klotho protein levels had the lowest SOD levels; meanwhile, SOD showed a positive correlation with RC and a negative correlation with α-Klotho. Given that α-Klotho protein displays antioxidant properties while RC promotes oxidative stress (13, 14), we speculate that individuals with higher α-Klotho protein may exist a lower oxidative stress response, thereby diminishing the requirement for antioxidant enzymes such as SOD. Conversely, lipid metabolism disorders induce oxidative stress, leading to a compensatory increase in SOD expression to counteract oxidative damage. These results suggest that elevated SOD may serve as an indirect marker of RC-related oxidative stress and lower α-Klotho levels. However, no significant association was found between RC and direct oxidative markers such as 8-OHdG, possibly due to measurement limitations or population-specific factors. Moreover, some studies have pointed out that overactive SOD may lead to the accumulation of hydrogen peroxide and induce secondary oxidative damage (41). Whether this paradoxical role of SOD also influences the relationship between RC and α-Klotho warrants further investigation.

Notably, while inflammation mediated the RC-LTL association and oxidative stress mediated the RC-α-Klotho link, we did not observe overlapping mediation effects across both biomarkers. This suggests that distinct biological pathways may differentially influence telomere shortening and α-Klotho reduction, reflecting heterogeneity in aging mechanisms. Future studies using broader biomarker panels and omics approaches are needed to clarify these divergent pathways.

To our knowledge, this is the first study to investigate the negative and nonlinear associations between RC and two critical aging biomarkers (LTL and serum α-Klotho). Meanwhile, it also provides novel insights into the mediating role of inflammation and oxidative stress in these associations, identifying TNFα and IL-6 as mediators in the negative relationship between RC and LTL, and SOD as a mediator in the adverse association between RC and α-Klotho protein. These findings highlight the unique value of RC, beyond traditional lipid markers, in linking dyslipidemia and biological aging, while supporting the involvement of inflammation and oxidative stress as potential mechanistic pathways.

However, several limitations should be acknowledged. First, the cross-sectional nature of the study precludes causal inference, underscoring the need for validation in prospective longitudinal cohorts. Second, the moderate sample size and the recruitment of participants from a single rural community may limit the external validity of our findings. Third, despite the adjustment of known confounders, unmeasured or unknown factors (such as dietary patterns, smoking, drinking, or genetic susceptibility) may still affect the results. Finally, the exploration of the mechanism in this study remains preliminary. Although five inflammatory and oxidative stress factors were detected, the differences between different aging markers were still not sufficiently elucidated. Additionally, we acknowledge that our mediation analyses were conducted using cross-sectional data and should be interpreted as statistical mediation only; temporal ordering cannot be established and residual confounding cannot be excluded. Future research should incorporate broader biomarker panels, longitudinal tracking, and multicenter cohorts to better clarify the role and mechanism of RC in aging.

## 5 Conclusion

This study revealed negative and nonlinear associations between RC and two aging biomarkers, LTL and serum α-Klotho, in the Chinese population, independent of traditional lipid parameters and multiple metabolic factors. Inflammation and oxidative stress may partly mediate these links. From an aging perspective, these cross-sectional findings suggest that greater attention to early RC management in addition to traditional lipids should be warranted, pending validation in longitudinal and interventional research.

## Data Availability

The original contributions presented in the study are included in the article/supplementary material. Further inquiries can be directed to the corresponding author.

## References

[1] Lublóy Á . Medical crowdfunding in a healthcare system with universal coverage: an exploratory study. BMC Public Health. (2020) 20:1672. doi: 10.1186/s12889-020-09693-3, PMID: 33167927 PMC7653851

[2] Zeng Q Gong Y Zhu N Shi Y Zhang C Qin L . Lipids and lipid metabolism in cellular senescence: Emerging targets for age-related diseases. Ageing Res Rev. (2024) 97:102294. doi: 10.1016/j.arr.2024.102294, PMID: 38583577

[3] Wadström BN Wulff AB Pedersen KM Jensen GB Nordestgaard BG . Elevated remnant cholesterol increases the risk of peripheral artery disease, myocardial infarction, and ischaemic stroke: a cohort-based study. Eur Heart J. (2022) 43:3258–69. doi: 10.1093/eurheartj/ehab705, PMID: 34661640

[4] Wadström BN Pedersen KM Wulff AB Nordestgaard BG . Elevated remnant cholesterol, plasma triglycerides, and cardiovascular and non-cardiovascular mortality. Eur Heart J. (2023) 44:1432–45. doi: 10.1093/eurheartj/ehac822, PMID: 36631967

[5] Huh JH Roh E Lee SJ Ihm SH Han KD Kang JG . Remnant cholesterol is an independent predictor of type 2 diabetes: A nationwide population-based cohort study. Diabetes Care. (2023) 46:305–12. doi: 10.2337/dc22-1550, PMID: 36469354

[6] Li L Lai J Zhang J Van Spall HGC Thabane L Lip GYH . Remnant cholesterol and risk of premature mortality: an analysis from a nationwide prospective cohort study. Eur Heart J Qual Care Clin Outcomes. (2024) 10:543–51. doi: 10.1093/ehjqcco/qcad071, PMID: 38100387

[7] Blasco MA . Telomeres and human disease: ageing, cancer and beyond. Nat Rev Genet. (2005) 6:611–22. doi: 10.1038/nrg1656, PMID: 16136653

[8] Dilixiati D Kadier K LaiHaiti D Lu JD Azhati B Rexiati M . Association between leucocyte telomere length and erectile dysfunction in US adults: a secondary study based on 2001–2002 NHANES data. BMJ Open. (2024) 14:e077808. doi: 10.1136/bmjopen-2023-077808, PMID: 38643009 PMC11033652

[9] Kuro-o M Matsumura Y Aizawa H Kawaguchi H Suga T Utsugi T . Mutation of the mouse klotho gene leads to a syndrome resembling ageing. Nature. (1997) 390:45–51. doi: 10.1038/36285, PMID: 9363890

[10] Kurosu H Yamamoto M Clark JD Pastor JV Nandi A Gurnani P . Suppression of aging in mice by the hormone Klotho. Science. (2005) 309:1829–33. doi: 10.1126/science.1112766, PMID: 16123266 PMC2536606

[11] Yamazaki Y Imura A Urakawa I Shimada T Murakami J Aono Y . Establishment of sandwich ELISA for soluble alpha-Klotho measurement: Age-dependent change of soluble alpha-Klotho levels in healthy subjects. Biochem Biophys Res Commun. (2010) 398:513–8. doi: 10.1016/j.bbrc.2010.06.110, PMID: 20599764 PMC4130489

[12] Liu Y Chen M . Emerging role of α-Klotho in energy metabolism and cardiometabolic diseases. Diabetes Metab Syndr. (2023) 17:102854. doi: 10.1016/j.dsx.2023.102854, PMID: 37722166

[13] Yang Z Ma Y Wang Y Jin M Bin J Chen Z . The prognostic value of serum α-klotho in age-related diseases among the US population: A prospective population-based cohort study. Prev Med Rep. (2024) 42:102730. doi: 10.1016/j.pmedr.2024.102730, PMID: 38689889 PMC11059319

[14] Kadier K Liu P Dilixiati D Peng X Ainiwaer A Kadier D . Maintaining ideal cardiovascular health is associated with higher serum anti-aging protein klotho in the middle-aged and older populations. J Nutr Health Aging. (2024) 28:100224. doi: 10.1016/j.jnha.2024.100224, PMID: 38582034 PMC12275714

[15] Varbo A Benn M Tybjærg-Hansen A Nordestgaard BG . Elevated remnant cholesterol causes both low-grade inflammation and ischemic heart disease, whereas elevated low-density lipoprotein cholesterol causes ischemic heart disease without inflammation. Circulation. (2013) 128:1298–309. doi: 10.1161/circulationaha.113.003008, PMID: 23926208

[16] Hu X Liu Q Guo X Wang W Yu B Liang B . The role of remnant cholesterol beyond low-density lipoprotein cholesterol in diabetes mellitus. Cardiovasc Diabetol. (2022) 21:117. doi: 10.1186/s12933-022-01554-0, PMID: 35761281 PMC9238255

[17] Bale BF Doneen AL Leimgruber PP Vigerust DJ . The critical issue linking lipids and inflammation: Clinical utility of stopping oxidative stress. Front Cardiovasc Med. (2022) 9:1042729. doi: 10.3389/fcvm.2022.1042729, PMID: 36439997 PMC9682196

[18] O'Donovan A Pantell MS Puterman E Dhabhar FS Blackburn EH Yaffe K . Cumulative inflammatory load is associated with short leukocyte telomere length in the Health, Aging and Body Composition Study. PloS One. (2011) 6:e19687. doi: 10.1371/journal.pone.0019687, PMID: 21602933 PMC3094351

[19] Armstrong E Boonekamp J . Does oxidative stress shorten telomeres *in vivo*? A meta-analysis. Ageing Res Rev. (2023) 85:101854. doi: 10.1016/j.arr.2023.101854, PMID: 36657619

[20] Li SS Sheng MJ Sun ZY Liang Y Yu LX Liu QF . Upstream and downstream regulators of Klotho expression in chronic kidney disease. Metabolism. (2023) 142:155530. doi: 10.1016/j.metabol.2023.155530, PMID: 36868370

[21] He S Wang N Tang Y Wang J Yin S Bai Y . Association between remnant cholesterol and anti-aging soluble α-klotho protein: New perspective on anti-aging from a NHANES study. Ir J Med Sci. (2024) 193:1249–51. doi: 10.1007/s11845-024-03640-6, PMID: 38366275

[22] Zhou M Zhu L Cui X Feng L Zhao X He S . Influence of diet on leukocyte telomere length, markers of inflammation and oxidative stress in individuals with varied glucose tolerance: a Chinese population study. Nutr J. (2016) 15:39. doi: 10.1186/s12937-016-0157-x, PMID: 27071648 PMC4830058

[23] XingB YuJ LiuY HeS ChenX LiZ . High dietary zinc intake is associated with shorter leukocyte telomere length, mediated by tumor necrosis factor-α: A study of China adults. J Nutr Health Aging. (2023) 27:904–10. doi: 10.1007/s12603-023-1992-z, PMID: 37960914

[24] Friedewald WT Levy RI Fredrickson DS . Estimation of the concentration of low-density lipoprotein cholesterol in plasma, without use of the preparative ultracentrifuge. Clin Chem. (1972) 18:499–502. doi: 10.1093/clinchem/18.6.499 4337382

[25] Cockcroft DW Gault MH . Prediction of creatinine clearance from serum creatinine. Nephron. (1976) 16:31–41. doi: 10.1159/000180580, PMID: 1244564

[26] Matthews DR Hosker JP Rudenski AS Naylor BA Treacher DF Turner RC . Homeostasis model assessment: insulin resistance and beta-cell function from fasting plasma glucose and insulin concentrations in man. Diabetologia. (1985) 28:412–9. doi: 10.1007/bf00280883, PMID: 3899825

[27] Liang G Schernhammer E Qi L Gao X De Vivo I Han J . Associations between rotating night shifts, sleep duration, and telomere length in women. PloS One. (2011) 6:e23462. doi: 10.1371/journal.pone.0023462, PMID: 21853136 PMC3154494

[28] Carreras-Badosa G Puerto-Carranza E Mas-Parés B Gómez-Vilarrubla A Gómez-Herrera B Díaz-Roldán F . Higher levels of serum α-Klotho are longitudinally associated with less central obesity in girls experiencing weight gain. Front Endocrinol (Lausanne). (2023) 14:1218949. doi: 10.3389/fendo.2023.1218949, PMID: 37522130 PMC10382686

[29] Hayes AF . Introduction to mediation, moderation, and conditional process analysis: a regression-based approach. New York, NY: The Guilford Press (2013).

[30] Loh NY Rosoff D Noordam R Christodoulides C . Investigating the impact of metabolic syndrome traits on telomere length: a Mendelian randomization study. Obes (Silver Spring). (2023) 31:2189–98. doi: 10.1002/oby.23810, PMID: 37415075 PMC10658743

[31] Yi SW Yi JJ Ohrr H . Total cholesterol and all-cause mortality by sex and age: a prospective cohort study among 12.8 million adults. Sci Rep. (2019) 9:1596. doi: 10.1038/s41598-018-38461-y, PMID: 30733566 PMC6367420

[32] Zhou L Wu Y Yu S Shen Y Ke C . Low-density lipoprotein cholesterol and all-cause mortality: findings from the China health and retirement longitudinal study. BMJ Open. (2020) 10:e036976. doi: 10.1136/bmjopen-2020-036976, PMID: 32801200 PMC7430481

[33] Ravnskov U Diamond DM Hama R Hamazaki T Hammarskjöld B Hynes N . Lack of an association or an inverse association between low-density-lipoprotein cholesterol and mortality in the elderly: a systematic review. BMJ Open. (2016) 6:e010401. doi: 10.1136/bmjopen-2015-010401, PMID: 27292972 PMC4908872

[34] Nordestgaard BG . Triglyceride-rich lipoproteins and atherosclerotic cardiovascular disease: new insights from epidemiology, genetics, and biology. Circ Res. (2016) 118:547–63. doi: 10.1161/circresaha.115.306249, PMID: 26892957

[35] Chen J Su Y Su X Luo F . Remnant cholesterol has a non-linear association with non-alcoholic fatty liver disease. Diabetes Res Clin Pract. (2023) 201:110733. doi: 10.1016/j.diabres.2023.110733, PMID: 37245725

[36] Wang Y Zha F Han Y Cai Y Chen M Yang C . Nonlinear connection between remnant cholesterol and stroke risk: evidence from the China health and retirement longitudinal study. Lipids Health Dis. (2023) 22:181. doi: 10.1186/s12944-023-01943-8, PMID: 37880769 PMC10601161

[37] Li J Lin Y . Association between residual cholesterol and sarcopenia in American adults. Front Endocrinol (Lausanne). (2024) 15:1461961. doi: 10.3389/fendo.2024.1461961, PMID: 39669500 PMC11634615

[38] Jurk D Wilson C Passos JF Oakley F Correia-Melo C Greaves L . Chronic inflammation induces telomere dysfunction and accelerates ageing in mice. Nat Commun. (2014) 2:4172. doi: 10.1038/ncomms5172, PMID: 24960204 PMC4090717

[39] Guo X Zhai Y Song C Mi Z Peng J Guo J . Elevated postprandial triglyceride-rich lipoproteins in patients with diabetes and stable coronary artery disease correlated with early renal damage and systemic inflammation. Lipids Health Dis. (2023) 22:58. doi: 10.1186/s12944-023-01820-4, PMID: 37138333 PMC10158000

[40] Yasui K Baba A . Therapeutic potential of superoxide dismutase (SOD) for resolution of inflammation. Inflammation Res. (2006) 55:359–63. doi: 10.1007/s00011-006-5195-y, PMID: 17122956

[41] Ekoue DN He C Diamond AM Bonini MG . Manganese superoxide dismutase and glutathione peroxidase-1 contribute to the rise and fall of mitochondrial reactive oxygen species which drive oncogenesis. Biochim Biophys Acta Bioenerg. (2017) 1858:628–32. doi: 10.1016/j.bbabio.2017.01.006, PMID: 28087256 PMC5689482

