# Correction: Remnant cholesterol shows inverse and nonlinear associations with leukocyte telomere length and serum α-Klotho, mediated by inflammation and oxidative stress

**DOI:** 10.3389/fendo.2025.1748130

**Published:** 2026-01-19

**Authors:** Baodi Xing, Jie Yu, Yiwen Liu, Qi Gao, Xinyue Chen, Shuli He, Fan Ping, Lingling Xu, Wei Li, Huabing Zhang, Yuxiu Li

**Affiliations:** 1 Department of Endocrinology, Key Laboratory of Endocrinology of National Health Commission, Translation Medicine Center, Peking Union Medical College Hospital, Chinese Academy of Medical Sciences and Peking Union Medical College, Beijing, China; 2 Department of Nutrition, Peking Union Medical College Hospital, Chinese Academy of Medical Sciences and Peking Union Medical College, Beijing, China

**Keywords:** remnant cholesterol, biological aging, leukocyte telomere length, serum α-Klotho, inflammation, oxidative stress

There was a mistake in **Table 2** as published. The regression coefficients for serum α-Klotho of models 3 and 4 in **Table 2** were wrong. The corrected **Table 2** appears below.

**Table 2:** The multiple linear regression between RC and LTL and serum α-klotho.

LTL	1mmol/L increment	Q1	Q2	Q3	Q4
model 1	-0.171(-0.236,-0.105)*	1(ref)	-0.061(-0.159,0.037)	-0.243(-0.342,-0.144)*	-0.263(-0.361,-0.164)*
model 2	-0.184(-0.269,-0.098)*	1(ref)	-0.038(-0.138,0.061)	-0.260(-0.362,-0.158)*	-0.235(-0.350,-0.120)*
model 3	-0.177(-0.262,-0.091)*	1(ref)	-0.038(-0.137,0.062)	-0.255(-0.358,-0.153)*	-0.230(-0.345,-0.114)*
model 4	-0.162(-0.250,-0.075)*	1(ref)	-0.033(-0.133,0.066)	-0.241(-0.345,-0.137)*	-0.217(-0.334,-0.010)*
α-Klotho	1mmol/L increment	Q1	Q2	Q3	Q4
model 1	-0.030(-0.046,-0.014)*	1(ref)	-0.032(-0.056,-0.007)*	-0.065(-0.090,-0.041)*	-0.054(-0.078,-0.029)*
model 2	-0.045(-0.066,-0.024)*	1(ref)	-0.030(-0.054,-0.005)*	-0.062(-0.087,-0.037)*	-0.058(-0.086,-0.030)*
model 3	-0.045(-0.066,-0.024)*	1(ref)	-0.030(-0.054,-0.005)*	-0.062(-0.087,-0.037)*	-0.058(-0.086,-0.030)*
model 4	-0.017(-0.045,0.011)	1(ref)	-0.023(-0.048,0.002)	-0.043(-0.073,-0.013)*	-0.034(-0.069,-0.001)*

Model 1 was adjusted for age and sex; model2 was adjusted for BMI, WHR, ALT, AST, eGFR, SBP, DBP, HbA1c, FBG, TG, HDL-C, LDL-C, and sUA; model3 was further adjusted for total energy intake based on model2; and model4 was further adjusted for TNF-α, IL-6, IL-1β, SOD, and 8-OHdG. The z-LTL and log-transformed α-klotho were analyzed in all models. *P < 0.05 means statistical difference.

There was a mistake in **Figure 2c** as published. There was a missing * for the total effect. The corrected **Figure 2** appears below. The original version of this article has been updated.

**Figure 2:**
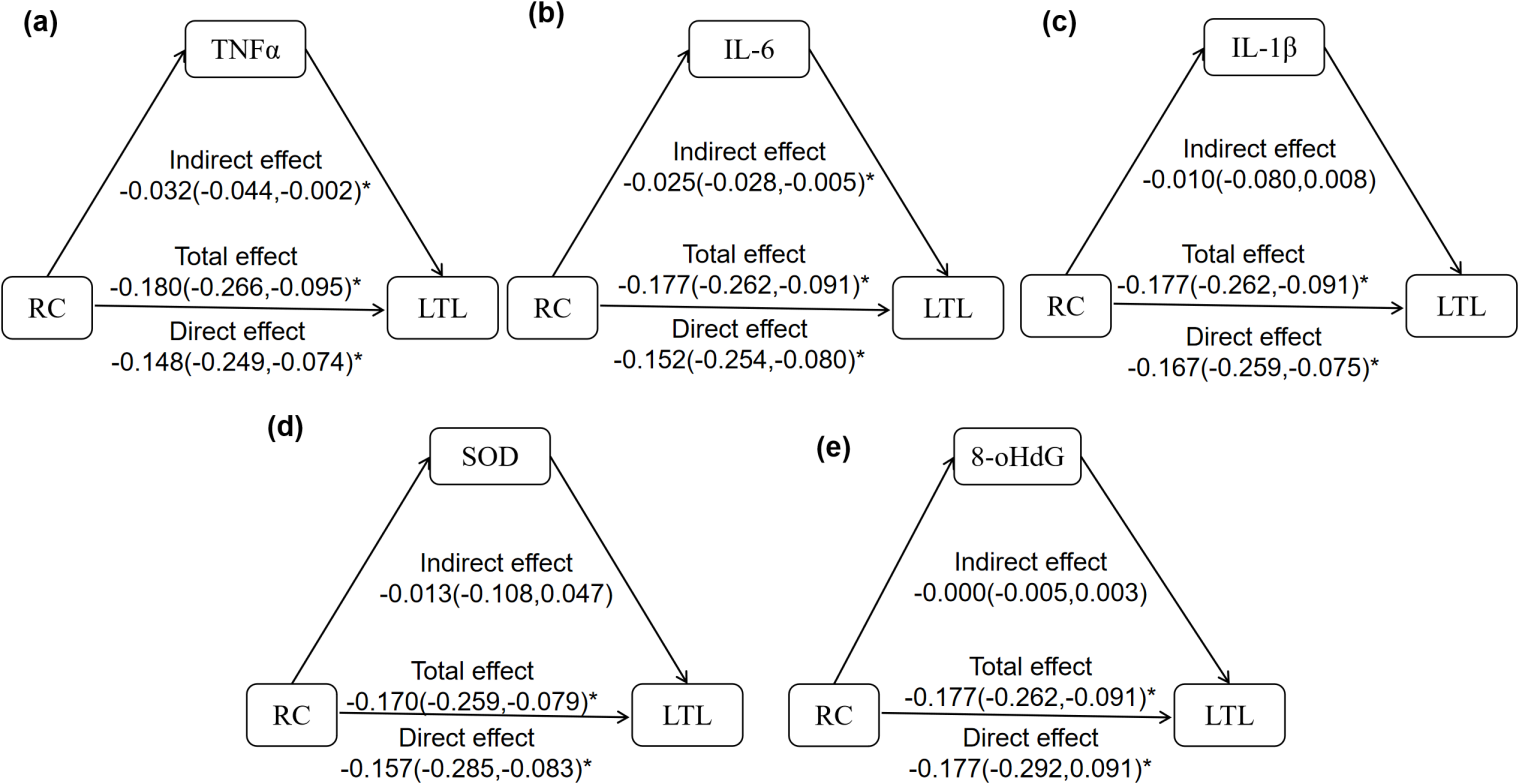
The mediation effect of inflammation and oxidative stress in the relationship between RC and LTL. **(a–e)** the mediation model of TNFα, IL-6, IL-1β, SOD, 8-OHdG on the correlation between RC and LTL. The model was adjusted for age, sex, BMI, WHR, ALT, AST, eGFR, SBP, DBP, HbA1c, FPG, LDL-C, HDL-C, TG, sUA, and total energy intake. The z-LTL and log-transformed TNFα, IL-6, IL-1β, SOD, and 8-OHdG were analyzed in the model. *P>0.05 means statistical difference..

