# Supplementary material for: Remnant cholesterol shows inverse and nonlinear associations with leukocyte telomere length and serum α-Klotho, mediated by inflammation and oxidative stress
Source: Front Endocrinol (Lausanne). 2025 Nov 17;16:1700349. doi: 10.3389/fendo.2025.1700349 (PMC12665542; doi:10.3389/fendo.2025.1700349)
Supplement: Supplementary file 1 [file DataSheet1.docx]

**Remnant Cholesterol Shows Inverse and Nonlinear Associations with Leukocyte Telomere Length and Serum α-Klotho, Mediated by Inflammation and Oxidative Stress**

**(Supplementary materials )**

**Page 2: Table S1** The numbers and percentages of missing covariate data

**Page 3: Table S2** Table S2.The association between different serum lipid indices and two aging biomarkers in univariate linear regression.

**Page 4: Table S3** Piecewise associations of RC defined by threshold with LTL and serum α-Klotho

**Table S1. The numbers and percentages of missing covariate data**

| Covirates | Numbers | Percentages (%) |
| --- | --- | --- |
| BMI | 1 | 0.01% |
| WHR | 20 | 1.90% |
| SBP | 5 | 0.48% |
| DBP | 5 | 0.48% |
| HbA1c | 1 | 0.01% |
| eGFR | 5 | 0.48% |
| TNFα | 24 | 2.28% |
| IL-6 | 28 | 2.66% |
| IL-1β | 21 | 2.00% |
| SOD | 12 | 1.14% |
| 8-OHdG | 13 | 1.24% |
| Energy | 92 | 8.75% |

**Table S2.The association between different serum lipid indices and two aging biomarkers in univariate linear regression.**

|  | LTL | | α-Klotho | |
| --- | --- | --- | --- | --- |
|  | β (95%CI) | *P* | β (95%CI) | *P* |
| TC | -0.079(-0.111,-0.046) | <0.001* | -0.007(-0.015,0.001) | 0.084 |
| LDL-C | -0.071(-0.117,-0.024) | 0.003* | -0.004(-0.016,0.007) | 0.486 |
| HDL-C | 0.062(-0.040,0.164) | 0.234 | 0.016(-0.009,0.041) | 0.197 |
| TG | -0.031(-0.051,-0.011) | 0.003* | 0.002(-0.003,0.007) | 0.484 |
| RC | -0.161(-0.226,-0.095) | <0.001* | -0.029(-0.045,-0.012) | <0.001* |

The z-LTL and log-transformed α-Klotho were analyzed in the model.**P*<0.05 means statistical difference.

**Table S3. Piecewise associations of RC defined by threshold with LTL and serum α-Klotho**

|  | β (95%CI) | *P* |
| --- | --- | --- |
| **LTL** |  |  |
| ≤0.645mmol/L | 0.054 (-0.449, 0.557) | 0.833 |
| 0.645-1.424mmol/L | -0.516 (-0.782,-0.250) | <0.001* |
| >1.424mmol/L | 0.291 (-0.050, 0.632) | 0.094 |
| **α-Klotho** |  |  |
| ≤1.374mmol/L | -0.062 (-0.093, -0.031) | <0.001* |
| >1.374mmol/L | 0.011(-0.065, 0.087) | 0.777 |

The z-LTL and log-transformed α-klotho were analyzed in the model.

The model was adjusted for age, sex, BMI, WHR, ALT, AST, eGFR, SBP, DBP, HbA1c, FPG, LDL-C, HDL-C, TG, sUA, and total energy intake. **P*<0.05 means statistical difference.
